# A Systematic Review and Meta-Analysis of Fecal Contamination and Inadequate Treatment of Packaged Water

**DOI:** 10.1371/journal.pone.0140899

**Published:** 2015-10-27

**Authors:** Ashley R. Williams, Robert E. S. Bain, Michael B. Fisher, Ryan Cronk, Emma R. Kelly, Jamie Bartram

**Affiliations:** 1 The Water Institute, University of North Carolina, Chapel Hill, NC, United States of America; 2 UNICEF, New York, NY, United States of America; Jinling Institute of Technology, CHINA

## Abstract

**Background:**

Packaged water products provide an increasingly important source of water for consumption. However, recent studies raise concerns over their safety.

**Objectives:**

To assess the microbial safety of packaged water, examine differences between regions, country incomes, packaged water types, and compare packaged water with other water sources.

**Methods:**

We performed a systematic review and meta-analysis. Articles published in English, French, Portuguese, Spanish and Turkish, with no date restrictions were identified from online databases and two previous reviews. Studies published before April 2014 that assessed packaged water for the presence of *Escherichia coli*, thermotolerant or total coliforms were included provided they tested at least ten samples or brands.

**Results:**

A total of 170 studies were included in the review. The majority of studies did not detect fecal indicator bacteria in packaged water (78/141). Compared to packaged water from upper-middle and high-income countries, packaged water from low and lower-middle-income countries was 4.6 (95% CI: 2.6–8.1) and 13.6 (95% CI: 6.9–26.7) times more likely to contain fecal indicator bacteria and total coliforms, respectively. Compared to all other packaged water types, water from small bottles was less likely to be contaminated with fecal indicator bacteria (OR = 0.32, 95%CI: 0.17–0.58) and total coliforms (OR = 0.10, 95%CI: 0.05, 0.22). Packaged water was less likely to contain fecal indicator bacteria (OR = 0.35, 95%CI: 0.20, 0.62) compared to other water sources used for consumption.

**Conclusions:**

Policymakers and regulators should recognize the potential benefits of packaged water in providing safer water for consumption at and away from home, especially for those who are otherwise unlikely to gain access to a reliable, safe water supply in the near future. To improve the quality of packaged water products they should be integrated into regulatory and monitoring frameworks.

## Introduction

The consumption of bottled water is a long-standing tradition in many European countries, and in recent decades consumption of bottled water has spread to other upper-middle and high-income countries (UM/HICs) for various reasons including taste, perceived health benefits, convenience, and concerns over the quality of municipal supplies [1,2]. The distrust of municipal supplies has led many consumers to purchase bottled water instead of drinking readily available tap water [3,4]. Some consumers have shifted from drinking other packaged beverages, such as soft drinks, to bottled water due to health concerns [5].

Growth in bottled water consumption has not been limited to UM/HICs. In many low-income and lower-middle-income countries (LICs) a growing number of households are turning to alternative water sources, including packaged water (PW), to meet their drinking water needs [6]. Use of improved drinking water sources has increased globally but according to the Joint Monitoring Programme (JMP) of WHO and UNICEF, 52% of people in developing regions do not have piped water on-premises, with the proportion rising to 82% in least developed countries (WHO/UNICEF 2014). These households collect water from sources outside the household, which results in a loss of time and energy [7]. At least 1.8 billion people use improved sources that are fecally contaminated [8,9]. The phrase ‘drinking water’ normally refers to water used for all domestic purposes including drinking, cooking, bathing, and laundry. However, only a fraction of domestic household water is consumed through drinking and cooking. We use the phrase ‘water for consumption’ to refer specifically to that fraction of drinking water that is consumed.

PW can provide a convenient alternative source of safe water for consumption for those households without continuous and easily accessible water supplies or those who would need to treat and/or carefully store water to ensure safety. Evidence suggests water stored in the home is significantly more contaminated than water at the source [10,11]. The sale of PW in shops, streets, schools, and workplaces offers consumers easy access to a water source for consumption outside the home. A study in Ibadan, Nigeria reported 88% of respondents traveled less than 100 meters to obtain PW [6]. In some areas where piped water is available, problems of intermittent service and water rationing lead households to purchase water from vendors [12]. Inadequate water infrastructure and lack of water access have led to the rapid development of the PW industry in many LICs.

PW is treated or untreated water for consumption that has been packaged in a container, such as a bottle or a plastic bag, which is then delivered to consumers or sold in stores or in the streets. Bottled water typically is divided into natural mineral waters and treated waters, depending on the source. Natural mineral waters originate from an underground source and are characterized by their mineral contents [13]. These are generally bottled at the source and typically do not undergo additional treatment, since they are considered to be free from pathogenic bacteria. PW other than natural mineral waters may come from various sources and are usually subjected to treatment processes by manufacturers to improve the quality of the water. Bottled water can be sold in individual-sized bottles (0.5–5 L) or in larger containers (5–20 L).

In West Africa, an early form of sachet water, referred to as ‘ice water’, consisted of 250–300 mL of water poured into a plastic bag that was hand-tied by the vendor and sold to consumers [6,14]. With the advent of relatively inexpensive turnkey processing machines, the industry largely transitioned from hand-filled bags to automated heat-sealed sachet machines. While larger PW companies have emerged in numerous countries, many producers are cottage industries operated out of apartments or small sheds [12].

With the expansion of the PW industry, some LIC national governments have attempted to regulate the industry, although many lack adequate regulatory and monitoring frameworks to ensure the safety of packaged drinking water products [15,16]. Even in countries with PW regulations, many unregistered producers are able to operate, due to the low barriers to entry and large potential profits [12]. While some manufacturers may adhere to stringent standards of quality, safety, and hygiene, others have been reported to sell untreated water from undeclared sources. Several recent studies have suggested that the microbial and chemical safety of PW products in LIC settings may not meet national or international guidelines [17–21]. In UM/HICs, a few recent studies have reported microbial contamination of PW [22–24]. Additional concerns have been raised regarding the environmental impacts of PW consumption including plastic waste generation and disposal especially given the limited recycling and municipal solid waste infrastructure in many LICs.

International guidelines for drinking water from the World Health Organization (WHO) recommend that water be free of detectable fecal indicator bacteria (FIB), specifically: *E*. *coli*, although thermotolerant coliforms (TTC) are considered an adequate substitute [25]. Other regulatory agencies use the concentration of total coliforms (TC) as a process indictor, in addition to FIB, with the presence of either TC or FIB in drinking water indicating the possible presence of pathogenic organisms due to a water treatment failure or contamination in the distribution system [26]. WHO and the Food and Agriculture Organization of the United Nations created the Codex Alimentarius Commission (CAC), which establishes international standards for food products. According to CAC, microbial standards for bottled water (with the exception of natural mineral waters) are set based on the most recent version of the WHO Guidelines for drinking water quality [27]. Additionally, the International Bottled Water Association (IBWA) publishes its own standards. The IBWA requires products to be free from detectable *E*. *coli* and TC in 100 mL of sample [28]. In the IBWA Code of Practice, if a final product tests positive for TC, then additional samples are taken and tested for TC and *E*. *coli*, with any subsequent positive samples resulting in a recall of the production lot [28].

Given the large burden of disease associated with inadequate provision of improved water services [29], it is reasonable to assume that the widespread use of PW has the potential to positively impact human health. However, the extent of the protective effect is unknown and is likely to be context-specific depending on the safety of other available water sources. As consumption of PW is increasing in both UM/HICs and LICs, it is important to understand the costs and benefits associated with PW consumption. Focusing on health aspects, the objectives of this study were to review the current literature on the microbial safety of PW, specifically with respect to FIB and TC. We used sub-group analyses to examine the following groups: <5 L bottles and sachets, machine-filled sachets versus hand-filled sachets, geographic regions; countries by income level; and PW and alternative water sources for consumption. Studies using longitudinal and cross-sectional sampling methods were included in the review.

## Methods

We conducted a systematic review of studies examining indicators of microbial quality of PW according to PRISMA guidelines for systematic reviews and meta-analysis (S1 Fig) [30]. The review protocol (S1 Text) was registered with PROSPERO (registration number CRD 42014007468).

### Inclusion criteria

In order to be eligible for inclusion in the review, studies must have examined the microbial quality of PW and reported findings in English, French, Portuguese, Spanish, or Turkish. PW was defined as water for consumption that is sold in a sealed container, either a sachet or bottle. Studies must have measured TC, TTC, and/or *E*. *coli* in at least 10 PW samples and reported the proportion of positive samples (or brands) as well as the total number of samples tested. Studies reporting fewer than 10 samples would likely lead to imprecise estimates of the proportion of samples containing FIB, and this exclusion criteria is consistent with other systematic reviews [11,31]. Studies were excluded if they were unclear which microbial parameter was examined, did not contain primary data, only examined carbonated or flavored PW, or if bacteria had been inoculated into PW. No restrictions were made based on study design or quality. We included both cross-sectional and longitudinal studies, whether or not the PW samples were selected at random, as well as studies whose primary aim was the evaluation of a water quality (diagnostic) test.

### Search strategy

We searched five online databases of peer-reviewed journals, African Index Medicus, Biosis Citation Index, Global Health Library, PubMed, and Web of Science without language restriction using ‘water’, terms related to PW, terms related to microbial water quality, and gastrointestinal health outcomes (for full search strategy see S1 Text). The search had no date restrictions. The results were restricted to peer-reviewed journal articles. Searches were conducted between January and April 2014. We identified additional relevant studies through searching the bibliographies of included studies and through Google scholar searches. Relevant papers identified from two previous reviews were also included [12,31].

### Eligibility Assessment

Two reviewers (ARW and ERK) independently screened the title and abstract of all identified studies. Full texts were obtained for any article identified as relevant by either reviewer and were examined by both reviewers to assess eligibility for inclusion in the review. A third reviewer (RC) resolved discrepancies at the full text stage. Additional reviewers assisted with full text reviews and data extraction of papers in French (RB), Portuguese (RB), Spanish (MF), and Turkish (AE). If the eligibility was unclear, authors were contacted to provide clarity.

### Data extraction

Data were extracted on: study characteristics (country, design, type of PW, total number of samples, total number of brands, sample collection site), study quality parameters (representativeness, randomization, quality assurance/quality control measures), and outcomes (proportion of samples positive for TC, TTC, and/or *E*. *coli*).

We extracted the number of samples positive for *E*. *coli*, and/or TTC and TC and the total number of samples tested. *E*. *coli*, TTC and TC were selected as indicators of contamination due to their widespread use and since they are recommended by international agencies including WHO [25]. When results were presented for various PW brands, we extracted data for each brand. In the case where studies collected water quality samples from other water sources used for consumption, additional data were extracted including the water source type, number of samples, and number of positive samples for each microbial parameter of interest. For studies examining the effects of storage, only the first set of measurements were extracted. Data were extracted for each PW type in studies that examined two or more PW types. Sachets were assumed to be machine-filled unless described as hand-filled. Bottled water was classified as small bottled water if the study did not state the volume.

Data were extracted by one reviewer (ARW) and entered into an extraction table created in Microsoft Excel. Data on study quality and bias were extracted during the data extraction step by one reviewer (ARW). As a quality control, 10% of studies in English (n = 17) were randomly selected for data extraction by a different reviewer (ERK).

### Analysis

Extracted data were exported to STATA IC/13.1 (StataCorp, College Station, TX) for random effects meta-regression and meta-analysis with logit transformed [32] proportions of positive samples (or brands) used as the outcome measure. A random effects model was used since heterogeneity in the results of the studies was anticipated. We identified sub-groups *a priori* in order to explore possible reasons for heterogeneity. The following subgroups were identified (a) bottled water versus sachet water; (b) machine-filled sachets versus hand-filled sachets; (c) location of PW samples along supply chain (manufacturer versus point of sale); (d) country income classification and (e) Millennium Development Goal (MDG) region. PW was classified according to the following types (i) small bottled water (<5 L), (ii) large bottled water (>5 L), (iii) dispensers (>5 L bottles installed on a dispenser), (iv) machine-filled sachets, and (v) hand-filled sachets. Included studies were classified as urban, peri-urban, rural, or national based on the description of the setting. Studies were also categorized by MDG region [33] and by national income level according to the 2014 World Bank classifications (low, lower middle, upper middle, high) [34]. *A posteriori* we explored differences in microbial quality between PW and other water sources used for consumption, the quality of which was reported in some included studies.

The variance of the transformed proportions was estimated using the inverse of the binomial variance, since it was rarely reported by studies. In order to account for proportions of 0 or 1, a continuity correction of 0.5 was employed for meta-regression [35]. Specifically: for studies in which no samples were positive, 0.5 was substituted for the number of positive samples. Similarly, for studies where all samples were positive, 0.5 was subtracted from the total number of positive samples, which was the denominator. The STATA *metareg* function was used for meta-regression. Prior to analysis, regional groups were combined as follows: Africa [North Africa, sub-Saharan Africa]; Asia [Southeast Asia, East Asia, South Asia, Oceania, West Asia]; Latin America. PW classification and regional grouping were used in meta-regressions.

To compare the quality of PW and other water sources, we performed meta-analysis using the STATA *metan* function and calculated random-effects (DerSimonian and Laird) pooled odds ratios of FIB contamination comparing any type of PW and all other water sources used for consumption within a given study. We conducted a sub-group analysis for studies that evaluated tap water in addition to PW. Since the sample sizes were unbalanced, a treatment arm continuity correction (TCC) factor for PW and control arm continuity correction (CCC) factor for other water sources were used in meta-analysis to account for proportions of 0 or 1 [35]. The heterogeneity of the included studies was assessed using the I^2^ test outlined by Higgins and Thompson [36]. The potential for bias from small study effects was assessed using funnel plots and Egger’s test [37]. Meta regression and meta-analysis were assessed at the 5% significance level. The results from studies that examined multiple PW types were combined during meta-analysis. Randomized and non-randomized studies were included in the meta-regression and the meta-analysis.

### Study quality

Articles were assigned a study quality score based on an equal weighting of criteria (one point each) outlined in Table 1 [31]. The impact of including lower quality studies (<4 points) in the review was examined by assessing the sensitivity of the results to exclusion of studies with less than four points.

**Table 1:** Criteria used to assess study quality (adapted from Bain et al. 2014b).

Criteria	Description
Representativeness	The study attempted to collect samples in a manner that was representative of the study area
Randomization	The samples were collected randomly
Randomization described	The method of randomization is clearly explained
Selection described	Description of how sampling sites were chosen (either PWMF or POS)
Specification of sample location	The location where samples were collected was clear (directly from manufacturers or point of sale)
Handling of samples described	The method of sample collection, handling, and timing of analysis after collection was described.
Proper sample handling	Reported transporting the samples at temperatures 2–6°C and analysis was performed within 6 hours
Method described	Clear explanation of the analytical method used to determine TC/FC/EC
Quality control measures	The use of duplicates (or triplicates), or positive/negative controls, or blanks was documented

### Assessment of bias

In order to assess bias within the studies, we compared the results of representative and non-representative studies, studies that included randomized sampling and those that did not, and compared longitudinal and cross-sectional studies. Longitudinal studies were defined as those that occurred over at least a six-month period with at least two samples collected at different times. We also performed a sensitivity analysis to determine the impact of combining TTC and *E*. *coli* as a single outcome measure (FIB).

## Results

### Search results

**Fig 1:**
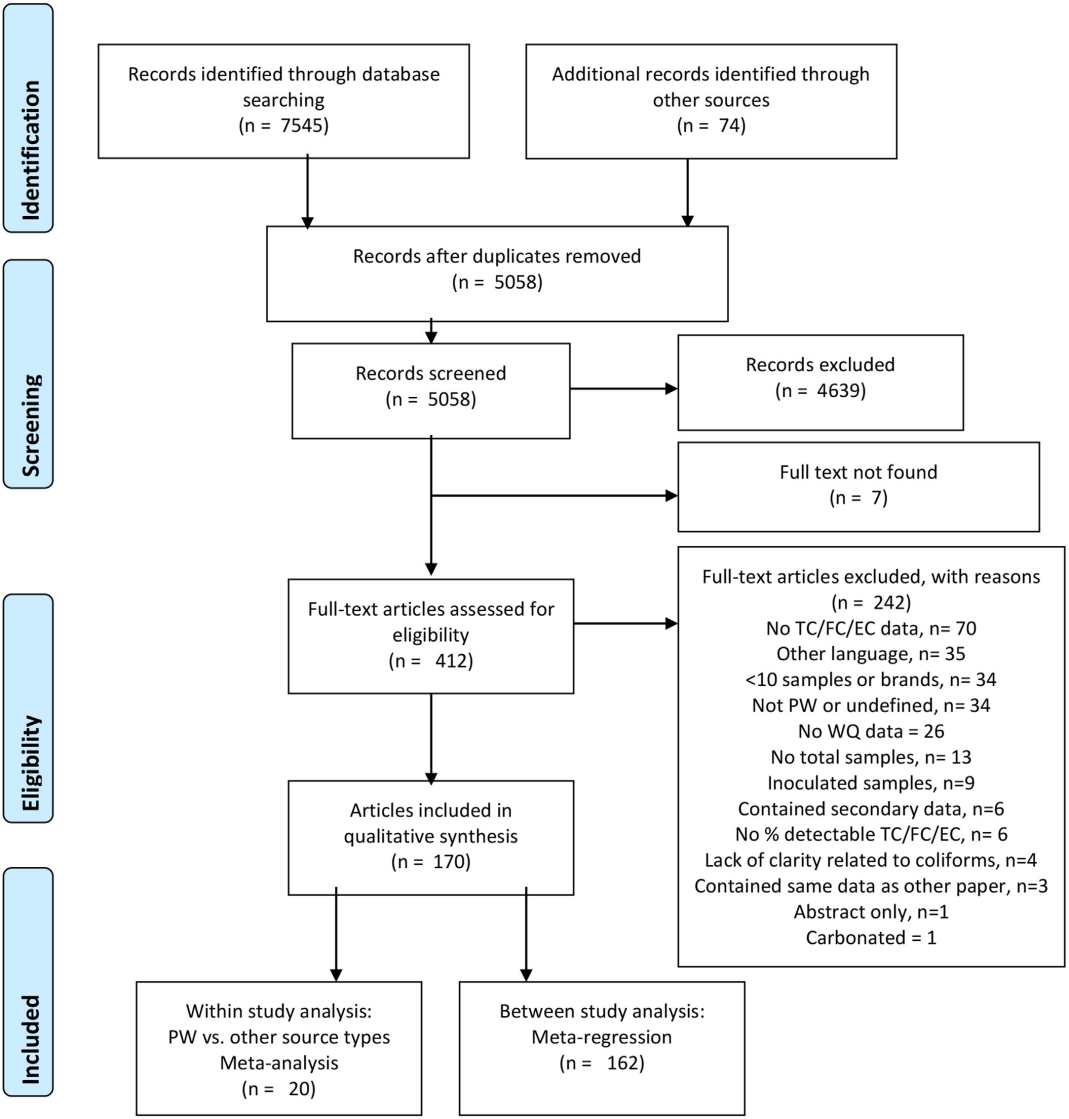
PRISMA flowchart. Results of literature search and screening according to PRISMA flowchart for systematic review screening process (Adapted from Moher et al. 2009).

A total of 7,545 articles were identified from the five databases and a further 74 articles were identified through bibliography searches, additional hand searching, and previous reviews (Fig 1). A total 4,639 studies were determined to be ineligible during title and abstract screening. At the full text review stage, 242 articles were excluded. The most common reasons for exclusion were that the study did not measure microbial indicators of interest (n = 70), was published in a language other than those included in the review (n = 35), or examined fewer than 10 samples (n = 34). Three studies were excluded because they contained the same dataset as another included paper [38–40]. The study with more complete information and data was included in the review. A total of 170 articles representing 172 distinct studies met the inclusion criteria [14,15,17,19,21–24,41–202].

### Study characteristics

**Fig 2:**
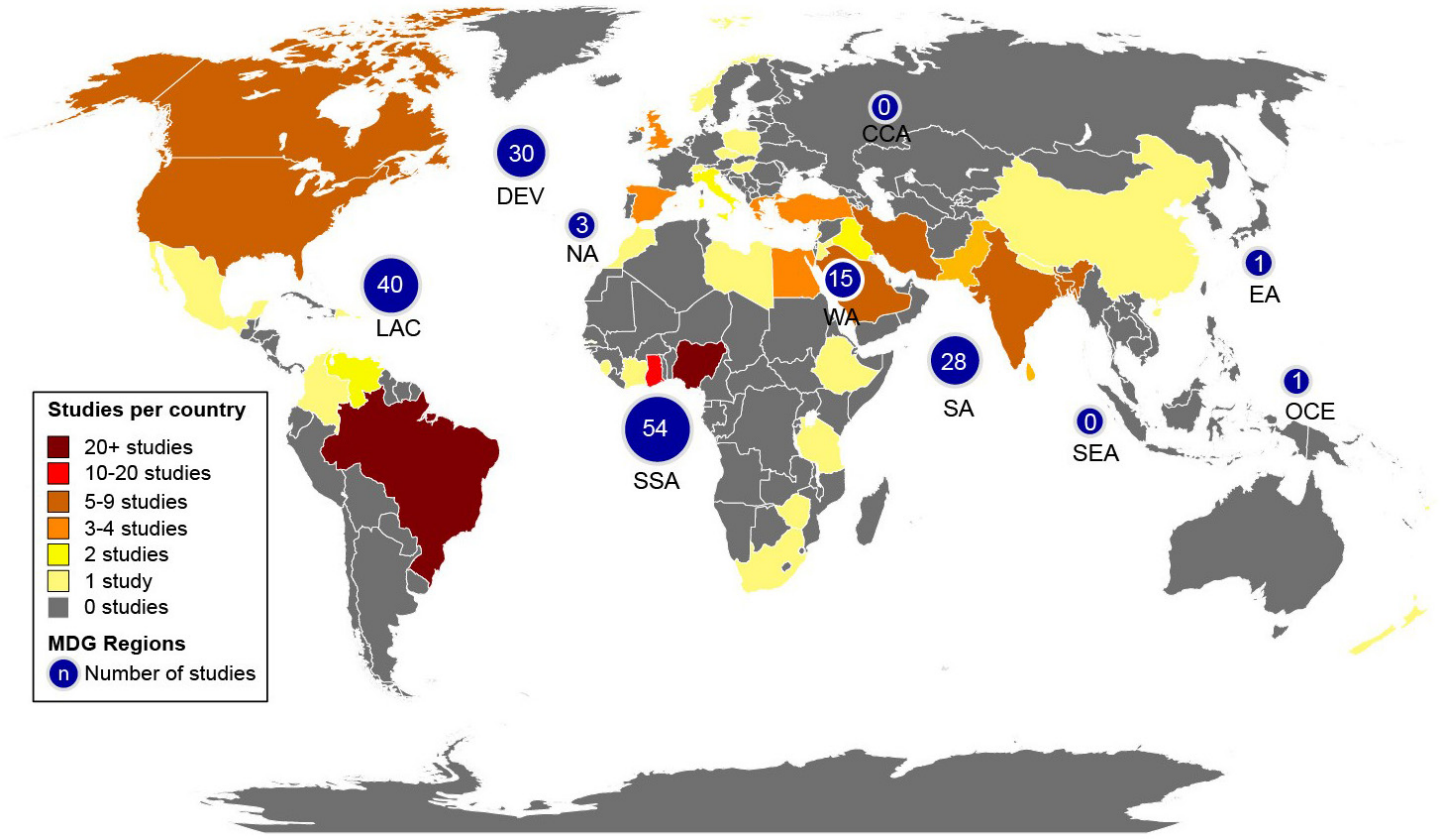
Geographic map of included studies. Distribution of included studies across Millennium Development Goal regions and country.

Table 2 summarizes the characteristics of included studies. The majority examined small bottles (n = 96, 56%), while 19% (n = 32), 4% (n = 7), 2% (n = 4), and <1% (n = 1), of studies examined machine-filled sachets, large bottles, dispensers, and hand-filled sachets, respectively. There were 23 (14%) studies that examined two types of PW and 5 (3%) studies that examined three types of PW. Two studies did not specify the type of PW product that was examined [70,170]. Of the included articles, 141 (82%) were published in English, 23 (13%), 6 (3%), 1 (<1%), and 1 (<1%) were published in Portuguese, Spanish, French, and Turkish, respectively. Most studies were from sub-Saharan Africa (31%) or Latin America (23%) (Fig 2). Very few studies were conducted in rural (3%, n = 5) [42,56,102,120,134] or peri-urban settings (2%, n = 4) [161,170,185,200].

**Table 2:** Characteristics of included studies.

	Number of studies (%)
PW type	
Sachet	49 (24%)
Sachet (hand-filled)	8 (4%)
Small bottles (0.5-<5L)	120 (60%)
Large bottles (5-20L)	15 (7%)
Dispensing	9 (4%)
Unspecified	2 (1%)
Point of sampling	
Manufacturers	16 (9%)
Point of sale	104 (60%)
Both Manufactures and POS	10 (6%)
Households/offices	7 (4%)
Study design^a^	
Cross-sectional	146 (85%)
Longitudinal	22 (13%)
Method (diagnostic)	3 (2%)
Randomized	58 (34%)
Representative	30 (17%)
Study quality	
Low (0–2)	69 (41%)
Medium (3–4)	77 (45%)
High (5–8)	24 (14%)
Language	
English	141 (82%)
French	1 (1%)
Portuguese	23 (13%)
Spanish	6 (3%)
Turkish	1 (1%)
Parameter	
*E*. *coli*	81 (47%)
Thermotolerant coliforms	84 (49%)
Total coliforms	155 (90%)
Sample size	
Small (10–30)	53 (31%)
Medium (31–100)	75 (44%)
Large (101–1941)	44 (25%)
Setting^a^	
National	14 (8%)
Urban	120 (70%)
Peri-Urban	3 (2%)
Rural	2 (1%)
Urban and Rural	3 (2%)
Urban and Peri-Urban	1 (<1%)
Unspecified/Regional	29 (17%)
Income Level^a^	
High income (HI)	36 (21%)
Upper-middle income (UM)	57 (33%)
Lower-middle income (LM)	69 (40%)
Lower income (LI)	10 (6%)
Total^a^	172

^a^Two articles reported two separate studies within the article, therefore the total is 172.

Cross-sectional studies accounted for the majority (n = 146, 85%) of study designs, with only 22 (12%) longitudinal studies included in the review. Five studies collected data in the same setting over multiple years [23,56,80,125,196]. One third (34%, n = 58) of studies reported randomized sample collection procedures, although very few (n = 11, 6%) of those studies described the method of randomization. No study met all nine criteria for study quality and only 14% (n = 29) of included studies received a score of five or higher (S2 Fig). Of the 37 studies examining the microbial quality of sachet water, 19% (n = 7) reported aseptically opening the sachet for analysis either by wiping the exterior of the sachet using ethanol, and/or cutting it using ethanol cleaned scissors, or using a syringe to extract the water from the interior.

Most studies (n = 104, 60%) reported collecting samples from the point of sale (POS), while only 9% (n = 16) of studies collected samples from manufacturers and 6% (n = 10) collected samples from both locations. A minority of studies did not report the location where samples were collected (20%, n = 34). For studies that identified the POS, 41% (n = 46) sampled from retail stores, 4% (n = 5) from street vendors, and 10% (n = 11) collected samples from both retail stores and street vendors.

Three studies examined the presence of contamination on the exterior of sachets [83,85,92]. Egwari et al. (2005) [83] found 45% of sample exteriors were contaminated with *E*. *coli*, however, the concentration of *E*. *coli* on the exterior was not associated with the concentration of *E*. *coli* in the water within the sachet. Ejechi and Ejechi (2008) [85] and Fisher et al. (2015) [92] also observed varying concentrations of contamination on the exteriors of sachets depending on the type of POS (e.g. retail store or street vendor). Both studies found a higher proportion of the exteriors of samples from street vendors to be contaminated with FIB and TC compared to the exteriors of samples from retail stores.

### PW quality by type and setting

Meta-regression was used to examine differences in the microbial quality of PW by type and setting (Table 3). Small bottled water was less likely to be contaminated with FIB and TC than other PW types (OR 0.32, 95%CI: 0.17–0.58, p<0.001; OR 0.10, 95% CI 0.05–0.22, p<0.001, respectively). Small bottled water was less likely to be positive for FIB and TC (OR 0.21, 95%CI: 0.10–0.42, p<0.001; OR 0.04, 95%CI: 0.02–0.09, p<0.001, respectively) compared to sachet water (machine-filled and hand-filled). Machine-filled sachets were also less frequently found to contain detectable FIB and TC than hand-filled sachets (OR 0.10, 95%CI: 0.02–0.53, p<0.01; OR 0.04, 95%CI: 0.01–0.26, p<0.001, respectively).

**Table 3:** Results from between-study meta-regression.

	Fecal indicator bacteria (>1 CFU/100mL)			Total coliforms (>1 CFU/100mL)		
Variables	Obs	OR (95% CI)	p-value	Obs	OR (95% CI)	p-value
PW type						
Small bottles vs. all other PW types	157	0.32 (0.17–0.58)	<0.001*	170	0.10 (0.05–0.22)	<0.001*
Small bottles vs. all sachets	137	0.21 (0.10–0.42)	<0.001*	147	0.04 (0.02–0.09)	<0.001*
Small bottles vs. machine- filled sachet	129	0.31 (0.16–0.62)	0.001*	139	0.08 (0.03–0.17)	<0.001*
Machine-filled sachet vs. hand-filled sachet	45	0.10 (0.02–0.53)	0.008*	44	0.04 (0.01–0.26)	0.001*
Study location						
LICs vs. UM/HICs	157	4.6 (2.6–8.1)	<0.001*	170	13.6 (6.9–26.7)	<0.001*
Africa vs. all other regions	157	3.0 (1.6–5.5)	0.001*	170	10.7 (5.1–22.6)	<0.001*
Asia vs. all other regions	157	1.7 (0.78–3.5)	0.192	170	0.94 (0.38–2.3)	0.889
South America vs. all other regions	157	0.71 (0.35–1.5)	0.350	170	0.40 (0.16–0.97)	0.044*
Developed vs. all other regions	157	0.12 (0.05–0.27)	<0.001*	170	0.08 (0.03–0.22)	<0.001*
Study characteristics						
Random vs. nonrandom	157	1.1 (0.59–2.2)	0.700	170	2.3 (1.0–5.2)	0.041*
Representative vs. non-representative	157	0.79 (0.36–1.7)	0.551	170	1.3 (0.47–3.5)	0.629
Longitudinal vs. cross-sectional	153	0.81 (0.32–2.0)	0.646	164	1.4 (0.38–5.2)	0.616
High quality vs. low-quality	157	0.99 (0.44–2.3)	0.988	170	2.1 (0.70–6.6)	0.178
*E*. *coli* vs. TTC	157	0.82 (0.44–1.5)	0.536			

*significance at 95%

With regards to national income classification, the results show a large difference in the quality of PW from LICs compared to products from UM/HICs with regards to FIB and TC (OR 4.6, 95% CI: 2.6–8.1, p<0.001; OR 13.6, 95%CI: 6.9–26.7, p<0.001, respectively). When only small bottles are considered, the difference remains significant for both FIB and TC (S1 Table, OR 3.4, 95%CI: 1.7–6.9, p<0.001; OR 6.0, 95%CI: 2.9–12.8, p<0.001, respectively).

Regionally, PW products from developed countries were significantly less likely to contain either FIB or TC compared to PW from all other regions (OR 0.12, 95%CI: 0.05–0.27, p<0.001; OR 0.08, 95%CI: 0.03–0.22, p<0.001, respectively). PW from Africa was 3.0 times (95%CI: 1.6–5.5, p<0.01) and 10.7 times (95%CI: 5.1–22.6, p<0.001) more likely to contain FIB and TC, respectively, compared to products from other regions. In contrast, PW from Latin America was less likely than PW from other regions to be contaminated with TC (OR 0.40, 95%CI: 0.16–0.97, p<0.001, respectively), although the results for FIB were not significant (p = 0.350). Furthermore, when examining only small bottles, only the difference between developed countries compared to all other regions remained significant for FIB and TC (S1 Table, OR 0.14, 95%CI: 0.06–0.30, p<0.001; OR 0.20, 95%CI: 0.08–0.48, p<0.001, respectively).

There were only 14 studies that collected both small bottles and sachet samples (excluding hand-filled sachets) (S3 Table). Of those, 12 measured FIB and only seven collected 10 or more samples of each PW type. There were six studies that collected samples of machine-filled sachets and hand-filled sachets, however one study reported results by brand and collected fewer than 10 brands [154].

### PW compared to alternative water sources for consumption

Twenty studies examined both PW and other water sources used for consumption, such as tap water, boreholes, hand pumps and/or surface water, within the same geographic area and with 10 or more samples. These studies provide insights into the microbial quality of the PW relative to other available sources of water for consumption.

**Fig 3:**
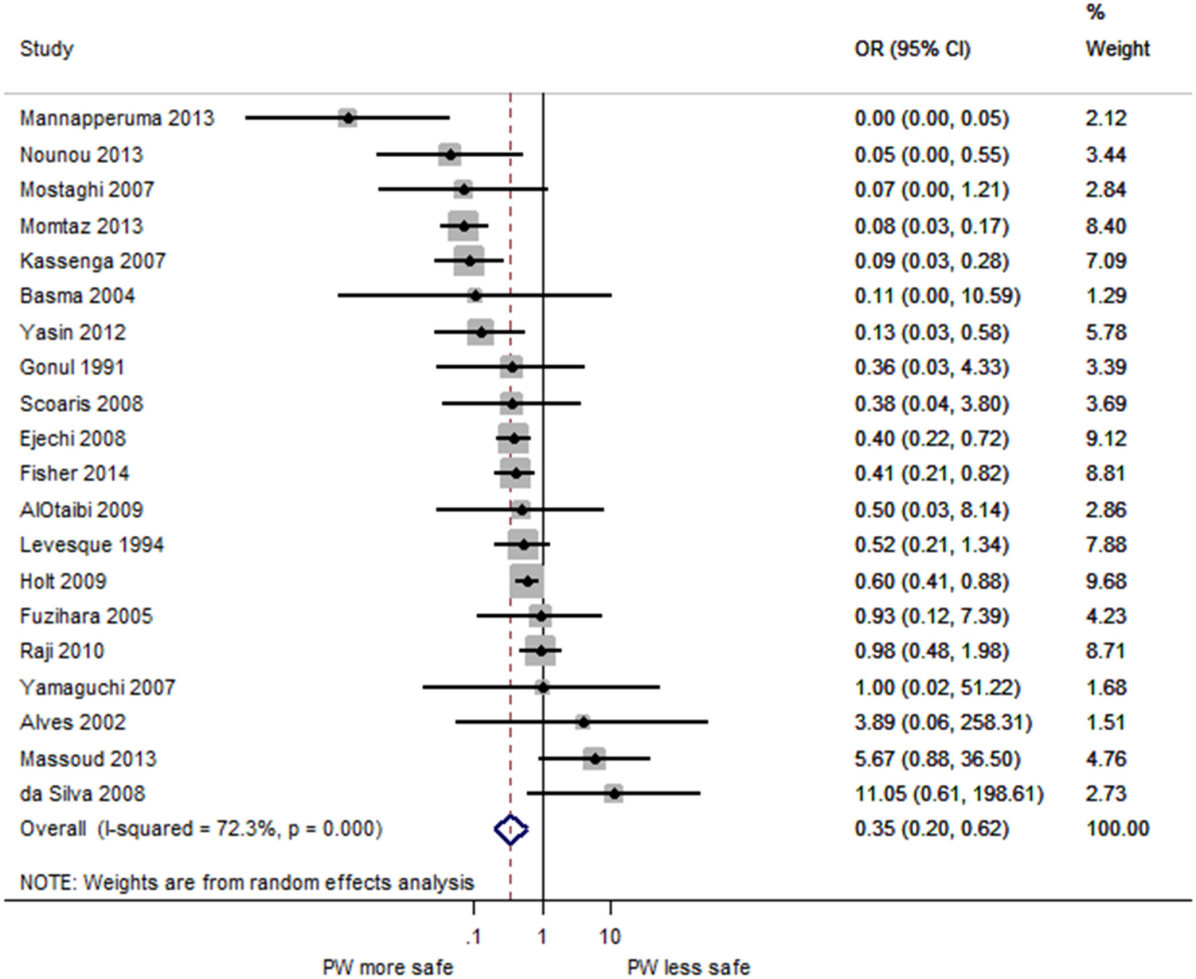
Forest plot of PW and all other drinking water sources. Forest plot of the odds ratio of fecal contamination comparing PW and all other drinking water sources.

Fig 3 is a forest plot of the odds of detectable FIB for PW compared to all other water sources used for consumption. PW was found to be significantly less likely to contain detectable FIB compared to other water sources used for consumption within the same area (pooled OR = 0.35, 95%CI: 0.20–0.62, p<0.001). The OR for some studies was greater than one, suggesting that PW may not always less contaminated compared to other water sources. The results vary considerably by study, as shown by the substantial heterogeneity between studies (I^2^ = 72.3%, p<0.001) [203].

**Fig 4:**
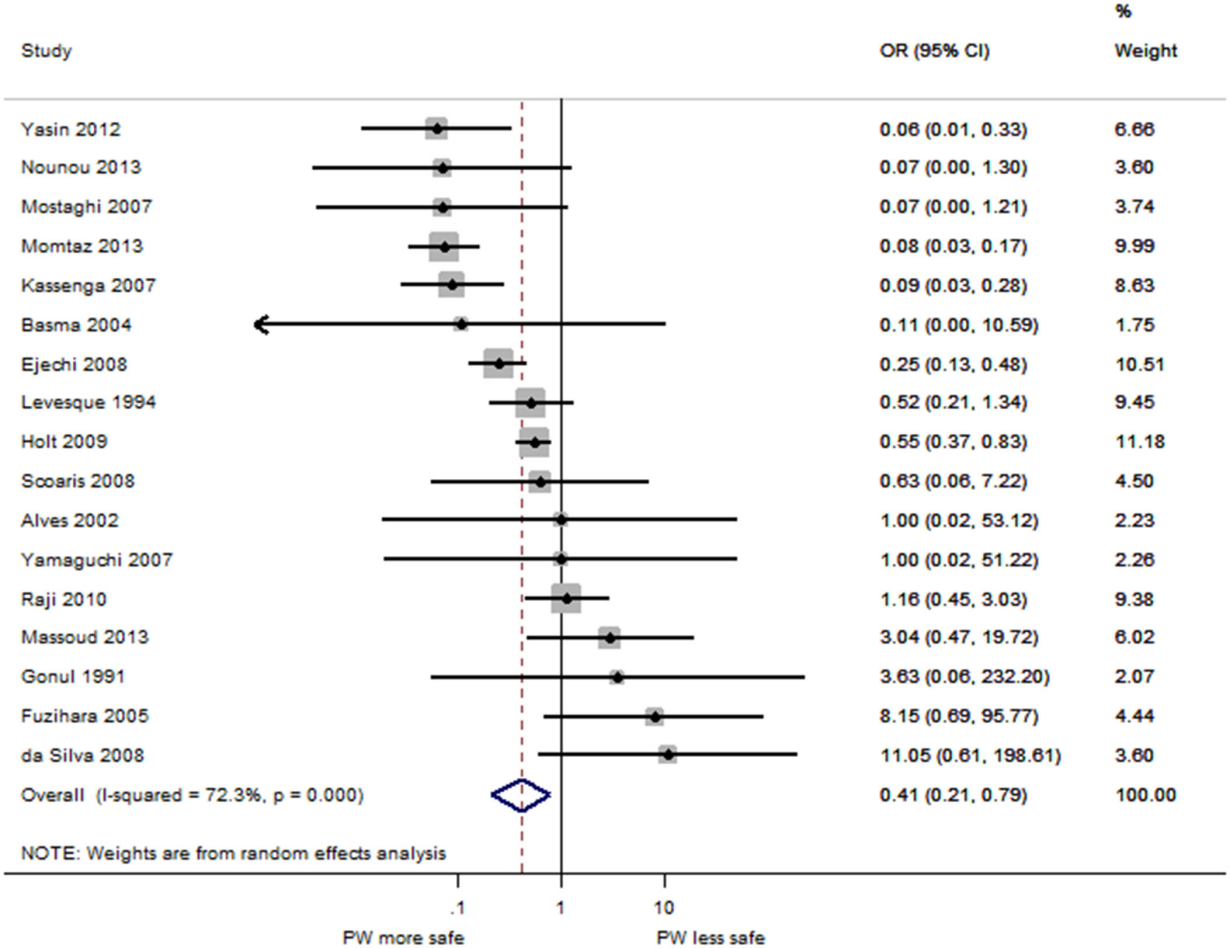
Forest plot of PW and tap water sources. Forest plot of the odds ratio of fecal contamination comparing PW and tap water sources.

The results of the meta-analysis comparing the OR for PW and tap water are shown in Fig 4. Overall, PW was significantly less likely to contain detectable FIB compared to tap water sources (pooled OR = 0.41, 95% CI 0.21–0.79, p<0.01). There was substantial heterogeneity across the studies (I^2^ = 72.3%, p<0.001) [203]. This could be due to differences in settings, analytical method, and/or season. Seven PW studies had calculated ORs greater than or equal to one, suggesting that while overall PW has a lower odds of contamination than tap water sources, this may not be the case in all study settings.

### Assessment of Bias

The results of Egger’s test for the meta-analysis of PW and all other water sources used for consumption and PW and tap water sources did not show evidence of small study effects (p = 0.461 and p = 0.756, respectively, S3 and S4 Figs). The results of the meta-regression showed no significant differences between high-quality and low-quality studies, or between representative and non-representative studies and cross-sectional and longitudinal studies, for both FIB and TC (Table 3). However, for TC, results of studies that randomized were significantly more likely to report contamination (p<0.05) compared to studies that did not use randomization. This finding remained significant when considering only small bottles (S1 Table). There was also no statistically significant difference in proportion of contaminated PW samples between studies that reported TTC or *E*. *coli* (p = 0.536).

## Discussion

A key finding from this review is that PW products are substantially less likely to be contaminated with FIB than alternative water sources for consumption, including tap water. In most studies PW was less likely to contain fecal contamination and may therefore be a safer source of water for consumption than other available sources with respect to microbial quality.

The results of the meta-regression illuminate the large disparity in the microbial quality of PW products between LICs and UM/HICs. PW products from LICs were 4.6 (95%CI: 2.6–8.1) times more likely to contain FIB and 13.6 (95% CI: 6.9–26.7) times more likely to contain TC compared to products from UM/HICs. The statistically significant difference between LICs and UM/HICs remains for both parameters when comparing only small bottles rather than all PW types. Given that many UM/HICs have a longer history with regulating and monitoring PW and packaged foods, the results may suggest that increased regulation and monitoring of PW products lead to an improvement in quality. As many LICs do not have effective regulatory structures in place for PW products, efforts to improve the ability of national governments to properly regulate and monitor these products may accelerate improvements in their safety [12,16]. The presence of TC in finished products indicates inadequate treatment and/or recontamination and suggests that current PW production and process control practices need to be improved in many LICs.

Similarly, PW products from developed nations were significantly less likely to contain FIB or TC compared to other geographic regions. PW products from sub-Saharan Africa were 3.0 (95%CI: 1.6–5.5) and 10.7 (95%CI: 5.1–22.6) times more likely to contain FIB and TC contamination, respectively, compared to other regions in aggregate. In contrast, PW from Latin America was statistically significantly less likely to contain TC than PW from other geographic regions in aggregate.

It is unclear whether there is a difference in PW quality between urban and rural settings, since so few studies have reported on PW quality in rural areas. Although PW industries are often located in urban regions, supply chains can deliver PW products from urban areas to remote rural settings. The change in safety of PW from urban points of manufacture to rural distribution areas has yet to be examined. Additionally, some small-scale PW producers are located in rural areas. In order to ensure PW products produced and distributed in rural areas meet national standards, it is advised that they be included in monitoring frameworks.

With the exception of one study from Colombia [194], the widespread consumption of sachet water is specific to LICs. Recent studies have reported the growth of the sachet water industry in areas of sub-Saharan Africa [12,16]. This review has shown that small bottled water is significantly less likely to contain FIB and TC compared to all other PW types in general and sachet water products in particular. In most places, bottled water is far more expensive per liter than sachet water [107,143]. The difference in quality between the two PW types may be a result of regulatory agencies focusing on bottled water since it is a more formal industry whereas sachet water is often more informal. Additionally, sachet water is more readily produced in small facilities that can rapidly relocate to avoid regulatory scrutiny or action [12]. Consumers of lower economic status who may not be able to purchase bottled water may therefore incur a greater risk of exposure to microbial contamination if they purchase other PW types. In a study of slum households in Accra, Ghana, sachet consumers tended to be of the poorest socio-economic level [204]. However, in contrast, data from recent Multiple Indicator Cluster Surveys (MICS) have demonstrated that the highest proportions of consumers primarily using sachet water were within the richest quintile in both Ghana and Nigeria [205,206]. Further work is needed to understand these apparently contradictory findings.

There is evidence that the quality of PW decreases along the supply chain [67,75,92,140], although few studies examined this in detail. Of the included studies, 10 examined the microbial quality of products along the supply chain and collected samples from manufacturers and POS. However, only six of these reported the results by sample location (at manufacturer or from POS) (S2 Table). The results of the six studies do not give conclusive evidence for differences in microbial quality with regards to FIB from manufacturers to POS, as none of the studies tracked PW products from the same batch and the results must be interpreted accordingly.

Sachet water presents an additional potential pathway of exposure due to the typical method of consumption in which consumers bite directly into the package. While only three studies examined exterior (packaging) contamination, all reported a proportion of exterior samples were positive for FIB. The exteriors of PW samples from street vendors were more frequently contaminated with TC compared to those of samples from retail stores [83,85,92]. While the impact of exterior contamination on health is unclear, it seems reasonable to recommend that sachets should not be removed from secondary packaging prior to sale in order to minimize exterior contamination.

A pervasive problem faced in data extraction was the inconsistent reporting of results according to brands or samples. Numerous studies reported collecting multiple samples per brand, however, results were then reported by brand rather than individual samples. Thus the results were biased upward, since one positive sample from a brand would be reported as a positive brand. Future research should clearly report all results according to sample and brand, where required.

Few studies compared small bottles to large bottles; however, the results from the meta-regression and qualitative study suggest that large bottles are more frequently contaminated with TC than smaller bottles. In many geographic regions, larger bottles are sold in 18–20 liter reusable containers while smaller bottled water is often sold in single-use containers. The higher prevalence of TC in large bottles may be a result of inadequate cleaning and disinfection of the reusable bottles at the manufacturing or refilling facility [24,122]. Although only a few studies examined larger bottles and dispensers, the review suggests that when bottles are installed on dispensers, additional contamination of both FIB and TC may occur. This could be due to improper maintenance of the dispenser including infrequent or ineffective cleaning [116]; however none of the studies reported a paired analysis of large bottles before and after installation. Consumer education about the proper use and cleaning of dispensers and improved design may help to reduce contamination of bottles.

PW provides a convenient source of water for consumption at home and also in settings outside the home. PW is often sold and consumed at restaurants, outdoor markets, in the streets [75,83], and near schools [114], in addition to mass gatherings such as sporting events or concerts. PW may also be available in workplaces [116,199], health care facilities, and emergency situations. A survey of PW consumers in urban Ibadan, Nigeria reported that 37% used sachet water outside the home [6]. International policymakers have recognized the importance of non-household settings and new water development goals and monitoring frameworks should include these settings [207–209]. The number of people exposed to unimproved water sources outside the home is unclear. Thus, PW may provide additional public health benefits as it could provide a safer alternative water source for consumption in non-household settings.

### Policy Implications

PW can provide safe water to large populations in diverse and in some cases under-served settings. Therefore, policymakers and regulators should acknowledge PW as an important source of water for consumption. However, current legislation and regulation of PW products and subsequent enforcement are imperfect, and as is the case with other sources of water for consumption, it has the potential to transmit waterborne diseases. Improvements to the legislative and regulatory frameworks surrounding PW products offer an opportunity for governments to capitalize on the potential public health benefits associated with PW.

The establishment of national standards for water quality, hygienic production, packaging, and distribution of PW products would set a minimum level of contamination that is acceptable and outline requirements for PW producers to meet. The CAC provides free standards and guidelines for water quality and hygienic production of bottled water that may be used as a starting point for policymakers interested in establishing national PW regulations. In areas where sachets are prevalent, they should be included in national regulations similar to bottled water and other vended products.

Sachets present distinct challenges to regulators compared to bottled water products. As the production of sachets can be easily performed by laypeople, especially if they are hand-filled, it can be difficult to track and ensure the quality of these products. Hand-filled sachets are typically produced without water treatment and using unhygienic methods with the result that many are frequently contaminated with FIB [146,147,154]. Therefore, in regions where hand-filled sachets are widely used officials should focus on eliminating their production and distribution.

As there is potential for disease transmission through PW, regulators should focus on improving their regulatory and monitoring systems to ensure public safety with regards to PW produced and/or distributed within their borders. The results suggest that monitoring should not only include sampling at the PW manufacturing facility but also at POS. Additional research is needed to examine degradation along the supply chain and elucidate the factors associated with contamination in order to inform effective regulation, control, and/or remediation.

As PW products are often more expensive than other sources per liter, there is an added economic burden on households relying on PW as their primary source of water for consumption. Since poorer households often purchase cheaper PW types (e.g. sachet water) that are often of lower quality, there are equity consequences to under-regulation. A recent study in Ibadan, Nigeria of consumer attitudes towards PW reported that 94% of respondents found the price of sachet water to be affordable [6]. It is unclear if additional regulation would result in PW producers increasing the cost of their products and thus create a barrier for lower income households to purchase PW [16].

As the results have suggested that PW may be a safer water source for consumption in some settings, regulators should seek to simultaneously improve the quality of PW in addition to other water sources used for consumption. The availability of PW does not eliminate the demand for piped water and/or other improved sources, as other domestic activities such as bathing and laundry require a larger volume of water, beyond what would be reasonable for households to obtain from PW. While the quality of water has health implications, there are also health effects related to the quantity of water available for hygiene purposes [210]. Therefore, while PW may be a safer water source for consumption, it cannot be a substitute for sufficient water supplies either at the household or community level.

### Limitations

This review included only a limited number of microbial quality parameters, TC, TTC, and *E*. *coli*. *E*. *coli* and TTC were chosen because they are recommended by the WHO as indicators of microbial drinking water quality, while TC was chosen because its presence in drinking water indicates inadequate disinfection [25]. While this is the first comprehensive review of microbial quality of PW, it does not explore other important indicator bacteria, such as enterococci, *Pseudomonas aeruginosa*, or other microbial contaminants such as protozoa, viruses, and helminths which are more resilient to many drinking water treatment and disinfection processes, such as chlorination [211]. PW products that are free from coliforms are not necessarily free from pathogens. A review of PW quality with respect to other key microbial indicators such as other bacteria, protozoa, viruses, helminths, and fungi would help provide a fuller picture of the microbial quality and safety of PW products.

Although PW standards may vary between countries, comparison of PW quality results to national guidelines was not within the scope of this review. In order to compare the microbial quality of PW from different countries, we used the guideline of no detectable *E*. *coli* in 100mL from the WHO Guidelines for Drinking Water Quality as the standard for comparison [25]. In certain countries, the results of some PW studies may be in compliance with national standards, although they would not meet the WHO guideline.

Natural mineral water is generally considered to be free from pathogenic organisms at the source; however, it does contain natural bacterial flora [212,213]. Regulations for the heterotrophic plate count (HPC) of natural mineral water often only require products to comply within 12 hours of bottling, however, previous research has shown HPC to increase after bottling [123]. Although the presence of HPC has not been associated with gastrointestinal illnesses, high counts can interfere with the detection of coliform bacteria using analytical methods that utilize lactose-based culture media [214]. Studies did not consistently report whether products were classified as natural mineral water, and therefore a comparison between mineral water and non-mineral water was not possible. Accordingly, some reported proportions of positive samples might have been underestimated if membrane filtration was used and high levels of HPC were present.

This review combines studies that used different analytical methods to detect TC and FIB. Differences in results may be due to the methods used, handling and transport of samples, hold times, randomization of sample collection, the use of quality control and quality assurance measures, and study settings. The study quality score used in this review was developed for studies of microbial water quality [31] but may not adequately reflect reliability of water quality assessments in individual studies as it is limited to reported information and equal weights were applied to the criteria. Studies reporting by brand instead of by sample could have overestimated the reported proportion of positive samples, since any one positive sample from one brand would result the brand being reported as positive. Additionally, some studies did not report the size of bottled water products analyzed, or if sachets were machine-filled or hand-filled, thus leading to possible misclassification with regards to PW type.

The search strategy may not have identified all relevant papers. It was designed to capture papers examining bottled and sachet water. However while screening papers, studies referred to larger >5 L bottles using other words such as ‘coolers’, ‘dispensers’ [65,116], or ‘demijohns’ [76], which were not included in the search strategy. In addition, search terms in different translations were not included in the strategy. While some papers examining larger bottled water products were captured through the search strategy, these additional names were not included and thus, this may not be an exhaustive review of large bottled water products.

Papers published in languages other than English, French, Portuguese, Spanish, or Turkish were excluded from this review, which may have introduced bias. During the screening phase, 35 papers published in other languages were identified through title and abstract as potentially relevant to the review, however the full text was not available in one of the five review languages.

Finally, comparison between geographic regions may have been confounded by the availability of studies from different countries (Fig 2), for example 71% of the included studies from Latin America were from Brazil, an upper-middle income country. Studies from Nigeria accounted for almost half (48%) of the studies from Africa.

## Conclusions

Consumers of PW products often perceive these products to be of higher quality than other water sources, since they assume that some form of additional water treatment has occurred. In the majority of studies, PW has been shown to be less likely to contain FIB and TC than other sources of water for consumption. As the PW industry continues to grow in LICs, it provides households with an option for safer water at and away from home, especially for those who are otherwise unlikely to gain access to a reliable, safe water supply in the near future.

The presence of FIB in finished PW products in several studies suggests the need for improved manufacturing processes, as well as improved regulation, monitoring, and enforcement. The inclusion of PW products along with other sources used for consumption in international, national, and local water quality monitoring frameworks would help to ensure potential benefits are realized.

This review demonstrates that small bottled water products are less likely to contain FIB and TC contamination than sachet water products, which are often of lower-cost. The convenience and price of sachet water enables many low-income households to obtain drinking water that may be safer than many other alternative sources. However, the disparity in quality between bottled and sachet water raises concerns over the equity of exposure to FIB among PW users. While PW is not a viable long-term solution as it is unable to meet household demand for domestic water quantity, in some contexts it may be the least unsafe source of water for consumption that is available to consumers. Therefore, the simultaneous improvement of the quality of both PW and municipal supplies and the continued expansion of improved water sources are recommended.

## Supporting Information

S1 Dataset Database of extracted data from included studies. (XLSX)

S1 Fig PRISMA Checklist for items to report for a systematic review and meta-analysis. (DOC)

S2 Fig Study quality. Study quality rating of included studies.
(DOCX)

S3 Fig Funnel plot of meta-analysis of PW and other drinking water sources. Egger’s funnel plot for meta-analysis of FIB contamination of PW and other drinking water sources.
(DOCX)

S4 Fig Funnel plot of meta-analysis of PW and tap water sources. Egger’s funnel plot for meta-analysis of FIB contamination of PW and tap water sources.
(DOCX)

S1 Table Results from studies examining the microbial quality of packaged water along the supply chain. (DOCX)

S2 Table Small bottled water analysis. Meta-regression for small bottled water samples only
(DOCX)

S3 Table Results comparing PW types within studies collecting data on more than one PW type. (DOCX)

S1 Text Systematic review protocol. Detailed description of systematic review protocol.
(DOCX)
