# Supplementary figures and images for: A Systematic Review and Meta-Analysis of Fecal Contamination and Inadequate Treatment of Packaged Water
Source: PLoS One. 2015 Oct 27;10(10):e0140899. doi: 10.1371/journal.pone.0140899 (PMC4624706; doi:10.1371/journal.pone.0140899)

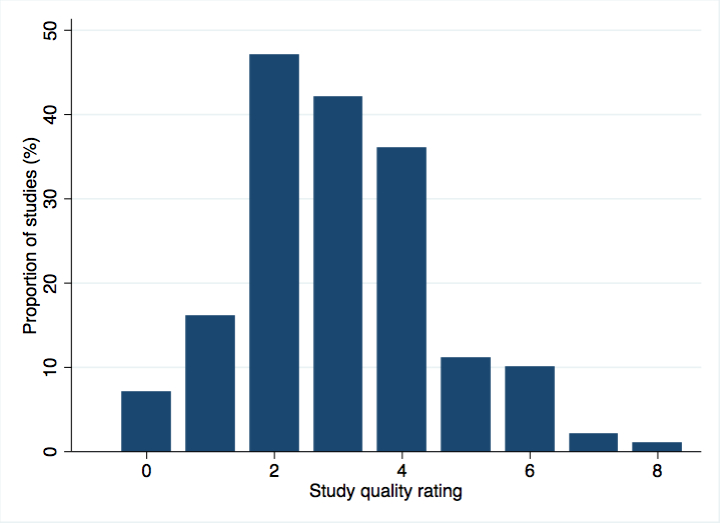


# S2 Fig. Study quality rating of included studies.

Supplement: S2 Fig — Study quality rating of included studies. (DOCX) [file pone.0140899.s003.docx]

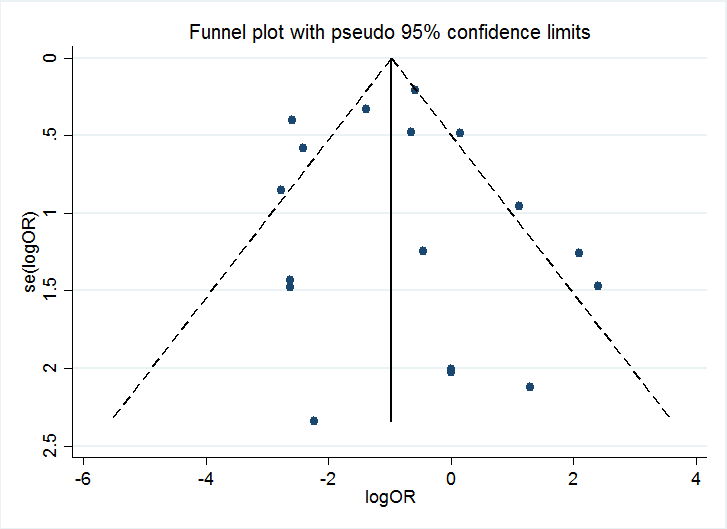


# S4 Fig. Egger’s funnel plot for meta-analysis of FIB contamination of PW and tap water sources.

Supplement: S4 Fig — Egger’s funnel plot for meta-analysis of FIB contamination of PW and tap water sources. (DOCX) [file pone.0140899.s005.docx]
