# Supplementary material for: A Systematic Review and Meta-Analysis of Fecal Contamination and Inadequate Treatment of Packaged Water
Source: PLoS One. 2015 Oct 27;10(10):e0140899. doi: 10.1371/journal.pone.0140899 (PMC4624706; doi:10.1371/journal.pone.0140899)
Supplement: S3 Fig — Egger’s funnel plot for meta-analysis of FIB contamination of PW and other drinking water sources. (DOCX) [file pone.0140899.s004.docx]

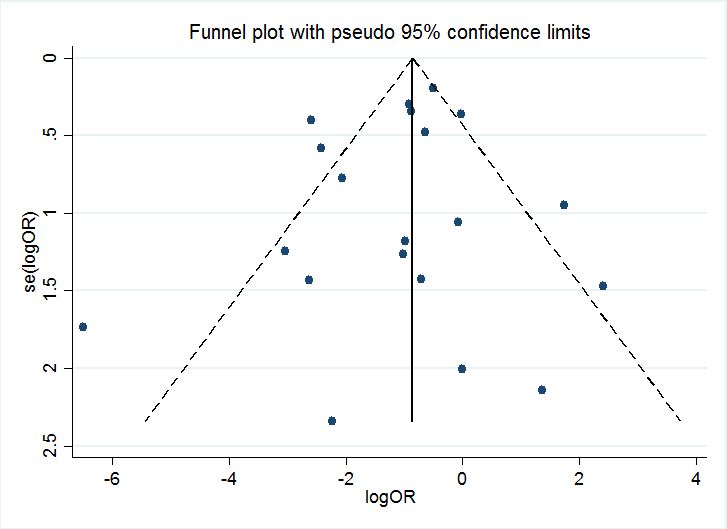


# S3 Fig. Egger’s funnel plot for meta-analysis of FIB contamination of PW and other drinking water sources.
