# Supplementary material for: A Systematic Review and Meta-Analysis of Fecal Contamination and Inadequate Treatment of Packaged Water
Source: PLoS One. 2015 Oct 27;10(10):e0140899. doi: 10.1371/journal.pone.0140899 (PMC4624706; doi:10.1371/journal.pone.0140899)
Supplement: S1 Table — (DOCX) [file pone.0140899.s006.docx]

# S1 Table. Meta-regression for small bottled water samples only.

|  | Fecal indicator bacteria  (>1 CFU/100mL) | | | Total coliforms  (>1 CFU/100mL) | | |
| --- | --- | --- | --- | --- | --- | --- |
|  | Obs | OR (95% CI) | p-value | Obs | OR (95% CI) | p-value |
|  |  |  |  |  |  |  |
| LICs vs. UM/HICs | 92 | 3.4 (1.7-6.9) | 0.001** | 103 | 6.0 (2.9-12.8) | <0.001** |
| Africa vs. all others | 92 | 0.86 (0.32-2.3) | 0.760 | 103 | 1.1 (0.36-3.5) | 0.834 |
| Latin America vs. all others | --- | --- | --- | 103 | 0.67 (0.27-1.7) | 0.389 |
| Developed vs. all others | 92 | 0.14 (0.06-0.30) | <0.001** | 103 | 0.20 (0.08-0.48) | <0.001** |
| Random vs. nonrandom | --- | --- | --- | 103 | 2.4 (1.1-5.4) | 0.036* |

*significance at 95%, **significance at 99%
