# Supplementary material for: A Systematic Review and Meta-Analysis of Fecal Contamination and Inadequate Treatment of Packaged Water
Source: PLoS One. 2015 Oct 27;10(10):e0140899. doi: 10.1371/journal.pone.0140899 (PMC4624706; doi:10.1371/journal.pone.0140899)
Supplement: S2 Table — Meta-regression for small bottled water samples only (DOCX) [file pone.0140899.s007.docx]

S2 Table. Results from studies examining the microbial quality of packaged water along the supply chain.

|  |  |  | Fecal indicator bacteria |  |  | Total coliforms |  |  |
| --- | --- | --- | --- | --- | --- | --- | --- | --- |
| Study | PW type | Total (N) | Manufacturer | Wholesaler | POS | Manufacturer | Wholesaler | POS |
|  |  |  | N positive (%) | N positive (%) | N positive (%) | N positive (%) | N positive (%) | N positive (%) |
| Akpoborie and Ehwarimo 2012 | SB^a^ & S | 27 | 0 (0%) | 0 (0%) | 0 (0%) | 0 (0%) | 0 (0%) | 0 (0%) |
| Biadglegne et al. 2009 | SB | 108 | 2 (6%) | 2 (6%) | 4 (11%) | 0 (0%) | 0 (0%) | 0 (0%) |
| Dada 2009 | S | 100 | 0 (0%) | 0 (0%) | 0 (0%) | 2 (7%) | 12 (40%) | 18 (45%) |
| Fisher et al. 2015 | S | 93 | 9 (19%) | --- | 17 (37%) | 18 (38%) | --- | 31 (67%) |
| Geldreich et al. 1975 | SB | 230 | 1 (2%) | --- | 0 (0%) | 6 (5%) | --- | 3 (0%) |
| Ohanu et al. 2012 | S | 5^b^ | 4 (80%) | --- | 2 (40%) | 3 (60%) | --- | 4 (80%) |

^a^SB – small bottles, S- sachets.

^b^number of brands reported rather than samples
