# Supplementary material for: A Systematic Review and Meta-Analysis of Fecal Contamination and Inadequate Treatment of Packaged Water
Source: PLoS One. 2015 Oct 27;10(10):e0140899. doi: 10.1371/journal.pone.0140899 (PMC4624706; doi:10.1371/journal.pone.0140899)
Supplement: S3 Table — (DOCX) [file pone.0140899.s008.docx]

**S3 Table. Results comparing PW types within studies collecting data on more than one PW type.**

|  | **Fecal indicator bacteria** | | | | **Total coliforms** | | | |
| --- | --- | --- | --- | --- | --- | --- | --- | --- |
| **Study** | **Total (N)** | **N positive (%)** | **Total (N)** | **N Positive (%)** | **Total (N)** | **N positive (%)** | **Total (N)** | **N Positive (%)** |
|  | **Small bottles** | | **Machine-filled sachets** | | **Small bottles** | | **Machine-filled sachets** | |
| Ajayi et al. 2008 | 10 | 0 (0%) | 78 | 0 (0%) | 10 | 0 (0%) | 78 | 4 (5%) |
| Akpoborie and Ehwarimo 2012 | 12 | 0 (0%) | 15 | 0 (0%) | 12 | 0 (0%) | 15 | 0 (0%) |
| Ampofo et al. 2007 | 17 | 0 (0%) | 179 | 15 (8%) | 17 | 0 (0%) | 179 | 72 (40%) |
| Danso-Boateng and Frimpong 2013 | 45 | 0 (0%) | 153 | 0 (0%) | 45 | 0 (0%) | 153 | 0 (0%) |
| Kassenga 2007 | 80 | 0 (0%) | 50 | 4 (8%) | 80 | 3 (4%) | 50 | 9 (18%) |
| Oyedeji et al. 2010 | 16 | 1 (6%) | 20 | 4 (20%) | 16 | 1 (1%) | 20 | 20 (100%) |
| Sumathy et al. 2004 | 11 | 0 (0%) | 10 | 0 (0%) | 11 | 0 (0%) | 10 | 0 (0%) |
|  | **Machine-filled sachets** | | **Hand-filled sachets** | | **Machine-filled sachets** | | **Hand-filled sachets** | |
| Ajayi et al. 2008 | 78 | 0 (0%) | 30 | 6 (20%) | 78 | 4 (5%) | 30 | 9 (30%) |
| Anunobi et al. 2006 | 12 | 0 (0%) | 48 | 8 (17%) | 12 | 10 (83%) | 48 | 47 (98%) |
| Obiri-Danso et al. 2003 | 88 | 2 (2%) | 40 | 9 (23%) | 88 | 4 (5%) | 40 | 17 (43%) |
| Okioga 2007 | 15 | 1 (7%) | 15 | 1 (7%) | 15 | 7 (47%) | 15 | 15 (100%) |
| Oloke 1997 | 15 | 13 (87%) | 29 | 28 (97%) | 15 | 15 (100%) | 29 | 29 (100%) |
|  | **Small bottles** | | **Large bottles** | | **Small bottles** | | **Large bottles** | |
| Alabdula'aly and Khan 1995 | --- | --- | --- | --- | 51 | 0 (0%) | 15 | 0 (0%) |
| Baumgartner and Grand 2006 | 36 | 0 (0%) | 12 | 0 (0%) | --- | --- | --- | --- |
| Falcone-Dias and Farache Filho 2013 | 264 | 0 (0%) | 60 | 0 (0%) | 264 | 0 (0%) | 60 | 0 (0%) |
| Falcone-Dias et al. 2012 | 179 | 0 (0%) | 84 | 2 (2%) | 179 | 2 (1%) | 84 | 13 (15%) |
| Rabee et al. 2012 | --- | --- | --- | --- | 30 | 1 (3%) | 12 | 3 (25%) |
| Robles et al. 1999 | 80 | 1 (1%) | 185 | 5 (3%) | --- | --- | --- | --- |
|  | **Large bottles** | | **Dispensers** | | **Large bottles** | | **Dispensers** | |
| Baumgartner and Grand 2006 | 12 | 0 (0%) | 174 | 0 (0%) | --- | --- | --- | --- |
| da Silva et al. 2008 | 22 | 0 (0%) | 77 | 5 (6%) | 22 | 5 (23%) | 77 | 31 (40%) |
| Lévesque et al. 1994 | 20 | 0 (0%) | 100 | 8 (8%) | 20 | 0 (0%) | 100 | 44 (44%) |
