# Supplementary material for: A Systematic Review and Meta-Analysis of Fecal Contamination and Inadequate Treatment of Packaged Water
Source: PLoS One. 2015 Oct 27;10(10):e0140899. doi: 10.1371/journal.pone.0140899 (PMC4624706; doi:10.1371/journal.pone.0140899)
Supplement: S1 Text — Detailed description of systematic review protocol. (DOCX) [file pone.0140899.s009.docx]

# S1 Text. Protocol for the systematic review.

Protocol for: A Systematic Review and Meta-Analysis of Fecal Contamination and Inadequate Treatment of Packaged Water

# **Background**

According to the WHO/UNICEF Joint Monitoring Programme (JMP), 748 million people lack access to an improved water source (WHO/UNICEF, 2014). This figure, however, does not include people who may have access to an improved supply but suffer from intermittent service delivery. In response to issues of reliability, adequate supply and an overall lack of access to piped water supplies there has been rapid growth in the packaged water industry, which includes bottled and sachet water (Dada 2011; Dada 2009). In addition to providing a convenient drinking water source, many consumers perceive packaged water to be of higher quality than other alternative drinking water sources.

However, as the industry has expanded, there has been a growing concern by health organizations over the quality of packaged water and its potential risk to public health. While countries with more established markets, such as those in Europe and North America, have already developed standards and regulations to safeguard consumers, regions with emerging packaged water markets are beginning to establish regulations for the industry. There have been numerous studies that have examined the microbial quality of packaged water with mixed results and conclusions (Addo et al. 2009; Ahmed et al. 2013; Anunobi et al. 2006; Ahmed et al. 2009).

In order to gain a better understanding of the microbial safety of packaged water, a systematic review was conducted to identify whether certain settings, national income levels, and packaging types have an influence on reported quality.

#

# **Objectives**

The objectives of this study are to review the literature on the microbial safety of packaged water and compare to WHO drinking water standards (WHO, 2011) to determine the potential risk to public health. Specifically the review will try to answer the following questions:

1. What is the quality of packaged water in terms of microbial parameters, specifically total coliforms, fecal coliforms, and *E. coli*, compared to the WHO guidelines for drinking water quality?
2. Are there differences in quality amongst various packaged water types?
3. What is the difference in quality of packaged water between countries of different income levels and between geographic regions?
4. What is the difference in quality of packaged water compared to other water sources used for consumption?

#

# **Methods**

## Search strategy

The review will be carried out according to the Preferred Reporting Items for Systematic Reviews and Meta-Analyses (PRISMA) guidelines and will be registered with Prospero. The following peer-reviewed databases, African Index Medicus, Biosis Citation Index, Global Health Library, PubMed, and Web of Science will be searched using the search strategy below.

(Water) AND

(Bottle OR Bottled OR Sachet OR “Packaged Drinking Water” OR “Packaged Water” OR “Packaged Beverages”) AND

(Quality OR Contaminant OR Contaminants OR Contaminate OR Contaminated OR Contamination OR Indicator OR Indicators OR Pathogen OR Pathogens OR pathogenic OR Bacteria OR Bacterial OR Bacteriological OR Microbe OR Microbes OR Microbial OR Microbiological OR Microorganism OR Microorganisms OR Organism OR Organisms OR Faecal OR Fecal OR “total coliform” OR coliform OR “Escherichia coli 0157 H7” OR “E. coli” OR Disease OR Morbidity OR Mortality OR Death OR Diarrhea OR Diarrhoea OR Dysentery OR Illness Or Gastroenteritis)

Adding the terms “package” or “packaged” to the search string results in too many articles about packaged food products, therefore we decided to only include the terms “packaged drinking water” and “packaged water”. Inclusion of the term “packaged” lacked specificity and additional abstracts from inclusion of this term were not found to be relevant. Sachet water is often referred to as “pure water,” however adding this phrase or the word “pure” to the search string also resulted in too many results and limited specificity. Therefore we rely on authors to use the terms sachet or packaged water in the title or abstract. There will be no language restrictions and no date restrictions, however each online database varies in terms of the earliest articles available online.

The bibliographies of included studies will be searched as well as papers from a previous systematic review (Bain et al. 2014) and papers identified by Stoler et al. (2012). Previously identified grey literature from Bain et al. (2014) related to packaged water will also be reviewed for inclusion in the review.

## Eligibility

### Studies will be included in the review based on the following criteria:

### They collect water samples of packaged drinking water; and

### Contain extractable results from primary water quality testing of at least one microbial parameter of interest (total coliforms, fecal coliforms, and *E. coli)*; and

1. Collect at least 10 samples of different sachets or bottles.

Only papers published in English, French, Portuguese, Spanish or Turkish will be included in the review. We will exclude studies that examine flavored or carbonated packaged water, are primarily a study related to perspectives and attitudes towards packaged water or are primarily concerned with water treatment methods for the production of packaged water products.

## Study selection

The results of each search from the peer-reviewed databases will be exported into EndNote. Once duplicates have been removed, two reviewers will independently screen the search results and exclude studies if they do not have meet the inclusion criteria based on a review of title and abstract. At the conclusion of abstract screening, the results from each reviewer will be exported into an Excel document where any discrepancies between the reviewers will be identified. Any study selected by either reviewer at the abstract stage will go through a full text review to determine if it should be included in the review.

## Data extraction

A data extraction table will be developed in Excel to facilitate the extraction process. The primary reviewer will extract basic information from the included studies such as title, author, journal of publication, and year of publication. Then characteristics of the study will be extracted such as country, region, setting (urban/rural), study design, sample selection, type of packaged water (bottled or sachet), size of the packaged water product, location of sample collection (manufacturing site, retail store, or street vendor), number of samples, number of brands, whether brands were domestic or imported, sample volume tested, parameters tested, and percentage of sample in compliance with WHO guidelines. Additional data will be extracted specific to the quality parameters that the study examined (such as mean, median, range, standard deviation, and variance). Additional information about quality control methods will be extracted such as handling and transportation methods, field blanks, field duplicates, and laboratory blanks.

As a quality control check, once the primary reviewer has completed the data extraction portion, 10% of the included studies will be randomly selected for the secondary reviewer to complete the data extraction table. The results from both reviewers will be compared to determine the variability in data extraction. A third co-author will handle discrepancies.

## Assessment of study quality

Included studies will be assessed in terms of quality based on the criteria listed in Table 1. Studies will not be excluded based on quality, however, differences in quality between studies will be assessed to determine their impact on the results.

## Analysis

Quantitative meta-analysis will be conducted using STATA IC/13.0 (StataCorp).

### Heterogeneity and sub-groups

### We will investigate the heterogeneity of the included studies using statistical methods outlined by Higgins and Thompson (2002). We will conduct sub-groups analyses to explore heterogeneity of the studies as well as answer our research objectives. We anticipate heterogeneity in the results of the studies and will seek to explore possible reasons for specific clustering of results. We have defined sub-groups and chose to use random effects before examining the data. The identified sub groups are: Bottled vs. sachet.

We will compare the results between studies with data on both sachet and bottles will be compared to determine differences between sachet and bottled water quality.

### Hand filled and machine filled sachets.

We will attempt to explore variations in sachet water quality between those filled by hand and those filled by machine. This will depend on how many included studies document the production process of the sachets.

### Location of packaged water products.

We will attempt to examine differences between the quality of packaged water products sold at retail stores and sold by street vendors. This will depend on the amount of included studies that clearly define the location where they collected samples and if they compared between retail settings within the study.

### Income levels and regions.

We will examine the differences in study results between countries of different income levels as defined by the World Bank income-classification. If there is enough data, we will also examine differences in results between geographic regions and urban and rural locations.

### Assessment of Bias

- Publication bias

Studies with negative findings could be published less frequently, thereby creating a publication bias. The extent of publication bias will be assessed using a funnel plot and Egger’s test for small study effects.

- Selection bias

We will attempt to explore possible selection bias in the studies based on the location of sample collection. Studies only collecting samples at the point of manufacture could underreport contamination. We will also examine how authors selected bottles and sachets to be sampled, whether they were randomly selected or if they attempted to have a representative sample of brands.

- Measurement bias

We will attempt to explore possible measurement bias related to the analytical method reportedly used by studies to analyze the water samples. Some studies may not have used standard methods that could result in an over or under-reporting of contamination. Specifically, we will examine their sample collection and handling procedures, if reported, and the sample volume used in analysis.

- Quality of study

Using the study quality score, we will assess whether lower quality studies have a tendency to report higher or lower levels of contamination.

#

# **Limitations**

This study may not identify all studies with data on packaged water quality, since only databases of peer-reviewed journal articles will be searched. However, we will review grey literature previously identified by Bain et al. (2014) for inclusion. An extensive examination of grey literature will not be a part of the search process.

# **Acknowledgements**

This review is funded by the Department for International Development. We would like to thank the Howard W. Odum Institute for Research in Social Science at the University of North Carolina, Chapel Hill for help with the statistical methods. We also thank Mellanye Lackey, MSI, at the Health Sciences Library at the University of North Carolina, Chapel Hill, for assistance in selecting search terms and electronic databases and in finding the full text of articles.

# **Conflict of Interest**

None declared.

# **References**

Addo KK, Mensah GI, Bekoe M, Bonsu C, Akyeh ML. 2009. Bacteriological quality of sachet water produced and sold in Teshie-Nungua suburbs of Accra, Ghana. Afr J Food Agric Nutr Dev 9(4):1019-1030.

Ahmad M, Bajahlan AS. 2009. Quality comparison of tap water vs. bottled water in the industrial city of Yanbu (Saudi Arabia). Environ Monit Assess 159:1–14; doi:10.1007/s10661-008-0608-8.

Ahmed W, Yusuf R, Hasan I, Ashraf W, Goonetilleke A, Toze S, et al. 2013. Fecal indicators and bacterial pathogens in bottled water from Dhaka, Bangladesh. Brazilian J Microbiol 44(1):97–103.

Anunobi CC, Onajole AT, Ogunnowo BE. 2006. Assessment of the quality of packaged water on sale in Onitsha Metropolis. Nig Ot J Hosp Med 16(2):56–59.

Bain R, Cronk R, Wright J, Yang H, Slaymaker T, Bartram J. 2014b. Fecal contamination of drinking-water in low- and middle-income countries: a systematic review and meta-analysis. PLoS Med 11(5):e1001644; doi:10.1371/journal.pmed.1001644.

Dada AC. 2011. Packaged water: optimizing local processes for sustainable water delivery in developing nations. Globalization and Health 7:24; doi:10.1186/1744-8603-7-24.

Dada AC. 2009. Sachet water phenomenon in Nigeria : Assessment of the potential health impacts. Afr J Microbiol Res 3(1):15–21.

Higgins JPT, Green S. 2011. Cochrane Handbook for Systematic Reviews of Interventions. Version 5.1.0. The Cochrane Collaboration. Available: [www.cochrane-handbook.org](http://www.cochrane-handbook.org) [accessed16 June 2014].

Higgins JPT, Thompson SG. 2002. Quantifying heterogeneity in a meta-analysis. Stat Med 21:1539–1558; doi:10.1002/sim.1186.

IBWA (International Bottled Water Association). 2012. Bottled Water Code of Practice. Alexandria, VA: IBWA. Available: http://www.bottledwater.org/files/IBWA-CODE-OF-PRACTICE-2012-FINAL.pdf [accessed 26 June 2013].

Joint Monitoring Program. (2013). Data resources and estimates-Introduction. Accessed 23 Jan. 2014. http://www.wssinfo.org/data-estimates/introduction/

Stoler J, Weeks JR, Fink G. 2012b. Sachet drinking water in Ghana’s Accra-Tema metropolitan area: past, present, and future. J Water Sanit Hyg Dev 2(4):223–240; doi:10.2166/washdev.2012.104.

WHO/UNICEF. 2014. Progress on Drinking Water and Sanitation: 2014. Update. Geneva: World Health Organization and UNICEF.

WHO (World Health Organization). 2011. Guidelines for drinking-water quality. 4^th^ ed. Geneva: World Health Organization.
